# Review on the Role of BRCA Mutations in Genomic Screening and Risk Stratification of Prostate Cancer

**DOI:** 10.3390/curroncol31030086

**Published:** 2024-02-22

**Authors:** Nikolaos Kalampokis, Christos Zabaftis, Theodoros Spinos, Markos Karavitakis, Ioannis Leotsakos, Ioannis Katafigiotis, Henk van der Poel, Nikolaos Grivas, Dionysios Mitropoulos

**Affiliations:** 1 Department of Urology, G. Hatzikosta General Hospital, 45001 Ioannina, Greece; kalampokas88@gmail.com; 2 Department of Laparoscopy and Endourology, Central Urology, Lefkos Stavros the Athens Clinic, PC 11528 Athens, Greece; zabaftisc@gmail.com (C.Z.); markoskaravitakis@yahoo.gr (M.K.); ioannisdleotsakos@gmail.com (I.L.); katafigiotis.giannis@gmail.com (I.K.); 3 Department of Urology, University of Patras Hospital, 26504 Patras, Greece; thspinos@otenet.gr; 4 Department of Urology, The Netherlands Cancer Institute-Antoni van Leeuwenhoek Hospital, 1066 CX Amsterdam, The Netherlands; h.vd.poel@nki.nl; 5 Department of Urology, Medical School, National & Kapodistrian University of Athens, 14122 Athens, Greece; dmitrop@med.uoa.gr

**Keywords:** prostate cancer, genomic screening, mutations

## Abstract

(1) Background: Somatic and germline alterations can be commonly found in prostate cancer (PCa) patients. The aim of our present study was to perform a comprehensive review of the current literature in order to examine the impact of BRCA mutations in the context of PCa as well as their significance as genetic biomarkers. (2) Methods: A narrative review of all the available literature was performed. Only “landmark” publications were included. (3) Results: Overall, the number of PCa patients who harbor a BRCA2 mutation range between 1.2% and 3.2%. However, BRCA2 and BRCA1 mutations are responsible for most cases of hereditary PCa, increasing the risk by 3–8.6 times and up to 4 times, respectively. These mutations are correlated with aggressive disease and poor prognosis. Gene testing should be offered to patients with metastatic PCa, those with 2–3 first-degree relatives with PCa, or those aged < 55 and with one close relative with breast (age ≤ 50 years) or invasive ovarian cancer. (4) Conclusions: The individualized assessment of BRCA mutations is an important tool for the risk stratification of PCa patients. It is also a population screening tool which can guide our risk assessment strategies and achieve better results for our patients and their families.

## 1. Introduction

Based on epidemiological data, prostate cancer (PCa) has been shown to be the most common of all urologic malignancies and second only to lung cancer as regards cancer-related mortality among male patients in developed countries [1]. The incidence of the disease has been steadily increasing over recent years [2], with the lifetime risk being equal to 12%, while the median age of diagnosis is 66 years according to the SEER database [3]. Although the widespread use of PSA as a screening tool has markedly increased the number of patients being diagnosed with occult disease [4], early detection has substantially decreased both cancer-specific mortality and advanced disease on initial diagnosis by 40% and 75%, respectively [5,6].

Commonly accepted risk factors for PCa include age, African race, several genetic factors and a family history of PCa [7]. More specifically, PCa is considered to be one of the malignancies with the greatest heritability component (up to 57% genetic contribution) [8], with several studies suggesting that 8–12% of all patients presenting with advanced disease may harbor a germline mutation in a tumor-suppressor gene [9,10]. Interestingly, it has been shown that individuals with a family history of PCa have 2.5 times higher probability of being diagnosed with the same disease when compared to the general population [11]. Researchers analyzing data form the Swedish Cancer Database concluded that the three malignancies with the highest familial cancer rate are prostate, breast and colorectal cancer (20.2% vs. 13.6% vs. 12.8%) [12]. An integrative analysis on patients diagnosed with advanced disease showed that somatic and germline alterations were found in up to 90% of those suffering from metastatic castration-resistant cancer [13]. So far, the most well-studied germline mutations relating to PCa are those of BRCA 1/2, CHEK2, NBM and ATM [10,14,15].

The main scope of the current review is to specifically examine the impact of BRCA mutations in the context of PCa, as well as their significance as a genetic biomarker in the era of efforts towards personalized screening. Emphasis is given to studies discussing prostate cancer risk stratification and early prostate cancer detection. Since our aim was to perform a narrative review, we included only “landmark” publications.

## 2. Role of Family History and BRCA Genes in PCa

In 2015, a study conducted by Liss et al. showed that male patients who had a history of PCa in their families were not only more susceptible to developing the same malignancy but they were also at a greater risk of dying from PCa, with both results being statistically significant. The same study highlighted the value of early PSA testing in family members of PCa patients, as it can lead to lower cancer-specific mortality rates and a possible survival benefit [16]. Likewise, a study performed by Brandt et al. showed that the hazard ratios for prostate cancer diagnosis and risk of death from prostate cancer in men increased with the number of affected relatives and decreased with increased age [17]. Also worth noting is the fact that we should treat familial and hereditary cancer as two distinct clinical entities. The hereditary form can be attributed to identifiable mutations (most commonly mutations regarding BRCA genes), while the familial form covers a larger spectrum (approximately 15–20% of PCa cases), with individuals having a positive family history in common but no identifiable genetic alteration. Nevertheless, the degree to which a positive family history defines the probability of developing cancer is highly variable and is also affected by factors such as age and degree of relation with the affected individuals. According to Carter et al., the proportion of PCa cases that could be attributed to mutated high-risk alleles was found to be approximately equal to 40% for men younger than 55 years, which is much higher than the 9% for individuals older than 85 years [18]. Moreover, a meta-analysis conducted by Zeeger et al. showed that men with an affected father had more than 50% lower relative risk of receiving a diagnosis of PCa than men with an affected brother (2.2 vs. 3.4), whereas this risk practically disappeared among second-degree family members [19]. Bratt et al. provided a nationwide population study, reporting the association between family history and diagnosis of prostate cancer in different cancer risk categories (any prostate cancer, non-low-risk prostate cancer and high-risk prostate cancer). According to them, the probabilities of intermediate and high-risk prostate cancer are better for counselling men with a positive family history of prostate cancer [20]. Having this in mind, it is easy to understand that in order to achieve a better estimation of the lifetime risk of being diagnosed with PCa, based solely on heritability factors, we should take into consideration the status of close relatives as well as the number of affected individuals in a family pedigree and their age at diagnosis. In this diagnostic algorithm, we should also take into account a family history from the maternal family branch [21].

BRCA 1 and BRCA 2 genes were first discovered almost three decades ago (1994 and 1995, respectively) after carefully examining the biological and genetic background of families with an abnormally high prevalence of breast and ovarian cancer [22,23]. These genes are responsible for 30–70% and approximately 90% of all hereditary breast and ovarian cancer cases, respectively. According to several studies, individuals harboring mutations in these genes have a lifetime risk of developing breast cancer of up to 85%, while the risk for ovarian cancer ranges from 20 to 40%. Individuals carrying BRCA1 mutations were also found to be at higher risk of developing other types of cancer, such as pancreatic and cervical. As far as prostate cancer is concerned, the risk was found to be age-dependent, affecting carriers younger than 65 years old [24,25].

In terms of biology, BRCA genes belong to the family of DNA damage repair (DDR) genes. The main role of these genes is to repair several DNA aberrations taking place during the cell cycle, thus providing genomic stability and ensuring an uneventful distribution of genetic material to the daughter cells following mitotic cell division [26]. In cases where this system fails, individuals become susceptible to malignancies such as breast, ovarian, prostate and pancreatic cancer. In particular, BRCA 1 mutations were associated with an increased risk of ovarian and breast cancer, whereas BRCA 2 carriers were more susceptible to pancreatic and prostate cancer [27]. According to several studies, the percentage of patients with PCa that harbor a BRCA2 mutation ranges between 1.2% and 3.2%, and the percentage is even smaller when it comes to BRCA1 [28,29]. Nevertheless, those genes (especially BRCA2) are considered to be responsible for most cases of hereditary PCa, with mutations in BRCA2 and BRCA1 increasing the risk by 3–8.6 times and up to 4 times, respectively, when compared to the general population [24,28,30,31].

So far, several studies have shown that PCa in individuals with mutated BRCA1/2 genes is in general more aggressive and associated with lower overall survival when compared to cases of male patients with normal alleles [32,33,34,35]. Back in 2019, Castro et al. conducted a retrospective analysis based on data from 79 BRCA mutation carriers and 1940 non-carriers, all of whom were diagnosed with PCa [33]. According to the authors, BRCA 1/2 mutations were found to be directly correlated with a higher risk of locally advanced disease, a higher Gleason score, nodal infiltration and distant metastases at the time of diagnosis. Moreover, they showed that BRCA2 mutations should be considered an independent negative prognostic factor based on both the significantly shorter 5-year cancer-specific survival and metastasis-free survival among mutation carriers.

Just to further examine the strength of the connection between BRCA mutations and PCa risk, Ibrahim et al. carried out a retrospective analysis by dividing a total of 102 men into two cohorts based on their genetic profile (mutation of either BRCA1 or BRCA2) [36]. Based on their findings, almost one-third of the under-examination individuals had at least one type of cancer, with prostate cancer being the most common (2 patients in the BRCA1 group and 11 patients in the BRCA2 group), followed by breast, skin and urothelial cancer. The above findings are in accordance with previous studies, supporting the theory that BRCA2 mutation carriers are far more susceptible to malignancies than BRCA1 carriers [25,30,37,38,39]. According to a well-designed case–control study on a special population of males of Ashkenazi origin, BRCA2 mutation carriers were shown to be at greater risk of developing PCa when compared to the age-matched control group [40]. Finally, according to the PROREPAIR-B trial, which prospectively examined only patients with metastatic castration-resistant disease, a germline BRCA2 mutation was characterized as an independent negative prognostic factor with a statistically significant lower CSS rate (17.4 vs. 33.2 months, *p* = 0.027). In this particular study, a statistically significant difference was also noticed in terms of treating BRCA2 carriers; namely, the use of ARTA (abiraterone or enzalutamide) was associated with better CSS and PFS in comparison with taxanes [41].

## 3. BRCA Testing in Prostate Cancer and Screening of Men with Known Mutations

Nowadays, it has become clear that PSA, which has been established as the major screening tool for PCa detection, has contributed to a small absolute decrease regarding the risk of death from PCa [42], while bringing with it the risk of overdiagnosis and the unnecessary treatment of quite a few individuals who would otherwise have never experienced clinical manifestations of the disease [43]. Under these circumstances, we can easily understand that a universal screening plan is related to a high financial burden as well as several unnecessary biopsies and treatment-related morbidities for individuals with indolent disease and a high PSA value [44,45]. On the other hand, we cannot overlook the importance of disease detection at an earlier stage, which gives us the opportunity to use a variety of therapeutic interventions with the aim of preventing cancer progression and metastatic disease [46]. Having all the above in mind, it is easy to justify the efforts made in recent years towards finding sufficient and objective data that could support the development of individualized screening plans. In the process of planning such strategies, we should take into consideration not only results from recent studies and population stratification tactics but also the need for shared and informed decision making.

In general, when we suspect the presence of an inherited malignancy or syndrome running through a family, it is of utmost importance that the patient is referred for genetic counseling. Unfortunately, in the case of PCa, there has been no uniform consensus so far regarding the exact criteria that should lead a patient to visit a geneticist.

In the context of PCa, without specific consideration of any particular genetic alteration, the American College of Medical Genetics suggests that a thorough genetic evaluation should take place if any of the following stands true [47]:

1. Two or more relatives receiving a PCa diagnosis at an age of 55 or younger (the relatives should be first-degree).
2. At least three first-degree relatives with PCa, irrespective of age.
3. Gleason grade 8 or higher and at least two individuals in the family pedigree diagnosed with breast, ovarian or pancreatic cancer.

Again, without specific interest for a specific mutation, the Johns Hopkins groups suggests another set of guidelines for familial PCa, which are as follows [48]:

1. Family pedigree with evidence of PCa in three successive generations.
2. Two relatives diagnosed with PCa at an early age (≤55 years).
3. At least three first-degree relatives with PCa.

Based on EAU recommendations, all individuals with high-risk or metastatic disease should be offered genetic counseling at least for the possibility that they have mutations in the BRCA1/2, ATM, FANCA or PALB2 genes. For the patients who are diagnosed with lower-risk disease, testing is recommended if any of the following are true [49]:

1. There is a strong family history of PCa.
2. At least one member of the family is diagnosed with Lynch syndrome.
3. There are already known germline mutations in the family or at least one family member has pancreatic or breast or ovarian cancer (possible BRCA2 mutation).

On the other hand, the National Comprehensive Cancer Network (NCCN) has published guidelines specifically for testing BRCA1/BRCA2 status, including patients with any of the following characteristics [50]:

1. Diagnosis of PCa (Gleason ≥ 7) and at least two relatives with prostate (Gleason ≥ 7) or breast or pancreatic cancer.
2. Metastatic PCa (proven with biopsy or imaging test).
3. Diagnosis of PCa (Gleason ≥ 7) at any age and at least one close relative with breast (age ≤ 50 years) or invasive ovarian cancer.

Serious efforts to reach a consensus and possibly bridge the gap between the recommendations made by different organizations have been made through studies like IMPACT. This study is an ongoing multicenter observational trial on carriers of BRCA1/2 mutations who have already been diagnosed with PCa [51]. The main purpose of the study is to determine the significance of an annual PSA testing protocol for BRCA1/2 carriers compared with non-carriers, with the PSA threshold for a biopsy being that of 3 ng/mL. In their interim analysis, researchers concluded that further follow-up is needed for BRCA1 carriers, but as regards the BRCA2 carriers, early and routine screening with PSA is important, as it was demonstrated that prostate biopsy in this specific population is associated with twice the positive predictive value compared to general population studies. An interim analysis of the IMPACT study showed that 77% of BRCA2 carriers were diagnosed with intermediate and high-risk disease [52]. A Dutch multidisciplinary expert panel reached a consensus in a meeting regarding the indications and applications of germline and tumor genetic testing in prostate cancer. Interestingly, they agreed that germline and tumor genetic testing should not be performed in the case of nonmetastatic hormone-sensitive prostate cancer when a relevant family history of cancer does not exist. As far as the metastatic disease is concerned, neither of the above-mentioned tests received panel approval for implementation in M1a HSPC, whereas it was agreed that the final therapeutic decision was not affected by the results. In metastatic CRPC, there was a lack of consensus regarding when tumor genetic testing should be performed and who should evaluate it, thus coming to no recommendations [53]. Table 1 summarizes the recommendations of different organizations for genetic testing in prostate cancer.

In an effort to establish risk-adapted guidelines for PCa diagnosis, the NCCN recommends that BRCA1/2 carriers should be tested annually starting at the age of 40 years [54]. The EAU follows the same directions with the exceptions of recommending intervals based upon the baseline PSA value and not including BRCA1 carriers in their guidelines [55]. Finally, the ACS does not take into consideration the germline status and suggests that men with a first-degree relative diagnosed younger than 65 years should start screening at 40 years (45 years if more than one first-degree relative with PCa) [56]. A recent review article by Giri et al. summarizes all existing guidelines for germline testing in prostate cancer, in an effort to promote multidisciplinary patient primary care. The authors also shed light on a very intriguing topic, namely the involvement of primary care physicians in the genetic testing process and algorithm. Through proper education, these professionals can offer individualized patient-centered medical advice and refer individuals with a positive family history at high risk for further evaluation and assessment by specialists [57].

## 4. Conclusions

Genetic studies during recent decades have undoubtedly added a lot to our current understanding of prostate cancer biology. Based on the current literature, it seems to be inevitable that genetic testing is very soon going to be an integral part of clinical practice not only for individuals already diagnosed with the disease but also in the setting of population screening. Hopefully, more precise knowledge on genetic predispositions for PCa is going to help us further improve our risk assessment strategies and achieve better results for our patients and their families.

## Figures and Tables

**Table 1:** Genetic testing recommendations for prostate cancer according to different organizations.

Organization	Gene Testing	Patient Characteristics
American College of Medical Genetics.	Thorough genetic evaluation.	Two or more first-degree relatives receiving a PCa diagnosis at an age of 55 or younger. At least three first-degree relatives with PCa, irrespective of age. Gleason grade 8 or higher and at least two individuals in the family pedigree diagnosed with breast, ovarian or pancreatic cancer.
Johns Hopkins groups.	Thorough genetic evaluation.	Family pedigree with evidence of PCa in three successive generations. Two relatives diagnosed with PCa at an age of 55 or younger. At least three first-degree relatives with PCa.
European Association of Urology (EAU) Recommendations.	Genetic counseling at least for the possibility that they have mutations in BRCA1/2, ATM, FANCA or PALB2 genes.	All individuals with high-risk or metastatic disease. For the patients who are diagnosed with lower-risk disease, testing is recommended if [49]: There is a strong family history of PCa. At least one member of the family is diagnosed with Lynch syndrome. There are already known germline mutations in the family or at least one family member has pancreatic or breast or ovarian cancer (possible BRCA2 mutation).
National Comprehensive Cancer Network (NCCN).	Specific BRCA1/BRCA2 status.	Diagnosis of PCa (Gleason ≥ 7) and at least two relatives with prostate (Gleason ≥ 7) or breast or pancreatic cancer. Metastatic PCa (proven with biopsy or imaging test). Diagnosis of PCa (Gleason ≥ 7) at any age and at least one close relative with breast (age ≤ 50 years) or invasive ovarian cancer.

## Data Availability

No new data were created.
